# Unraveling the Mechanism of the Endophytic Bacterial Strain *Pseudomonas oryzihabitans* GDW1 in Enhancing Tomato Plant Growth Through Modulation of the Host Transcriptome and Bacteriome

**DOI:** 10.3390/ijms26051922

**Published:** 2025-02-23

**Authors:** Waqar Ahmed, Yan Wang, Wenxia Ji, Songsong Liu, Shun Zhou, Jidong Pan, Zhiguang Li, Fusheng Wang, Xinrong Wang

**Affiliations:** Guangdong Province Key Laboratory of Microbial Signals and Disease Control, College of Plant Protection, South China Agricultural University, Guangzhou 510642, China; ahmed.waqar1083@yahoo.com (W.A.); wangyan120821@163.com (Y.W.); jwx0106@163.com (W.J.); liusongsong0606@163.com (S.L.); zhoushun173@163.com (S.Z.); pjd5403@163.com (J.P.); lizhiguang250106@163.com (Z.L.); wfsscau@163.com (F.W.)

**Keywords:** *Pseudomonas oryzihabitans*, tomato plant growth, plant–microbe interactions, multiomics analyses, sustainable agriculture

## Abstract

Endophytic *Pseudomonas* species from agricultural crops have been extensively studied for their plant-growth-promoting (PGP) potential, but little is known about their PGP potential when isolated from perennial trees. This study investigated the plant-growth-promoting (PGP) potential of an endophyte, *Pseudomonas oryzihabitans* GDW1, isolated from a healthy pine tree by taking tomato as a host plant. We employed multiomics approaches (transcriptome and bacteriome analyses) to elucidate the underlying PGP mechanisms of GDW1. The results of greenhouse experiments revealed that the application of GDW1 significantly improved tomato plant growth, increasing shoot length, root length, fresh weight, and biomass accumulation by up to 44%, 38%, 54%, and 59%, respectively, compared with control. Transcriptomic analysis revealed 1158 differentially expressed genes significantly enriched in the plant hormone signaling (auxin, gibberellin, and cytokinin) and stress response (plant–pathogen interaction, MAPK signaling pathway-plant, and phenylpropanoid biosynthesis) pathways. Protein–protein interaction network analysis revealed nine hub genes (*MAPK10*, *ARF19-1*, *SlCKX1*, *GA2ox2*, *PAL5*, *SlWRKY37*, *GH3.6*, *XTH3*, and *NML1*) related to stress tolerance, hormone control, and plant defense. Analysis of the tomato root bacteriome through 16S rRNA gene amplicon sequencing revealed that GDW1 inoculation dramatically altered the root bacterial community structure, enhancing the diversity and abundance of beneficial taxa (Proteobacteria and Bacteroidota). Co-occurrence network analysis showed a complex bacterial network in treated plants, suggesting increasingly intricate microbial relationships and improved nutrient absorption. Additionally, FAPROTAX and PICRUSt2 functional prediction analyses suggested the role of GDW1 in nitrogen cycling, organic matter degradation, plant growth promotion, and stress resistance. In conclusion, this study provides novel insights into the symbiotic relationship between *P. oryzihabitans* GDW1 and tomato plants, highlighting its potential as a biofertilizer for sustainable agriculture and a means of reducing the reliance on agrochemicals.

## 1. Introduction

Tomato (*Solanum lycopersicum*) is an economically important vegetable crop worldwide and is famous for its nutritional value and versatility in food production [1]. China is acknowledged as the foremost producer of tomatoes worldwide, producing 68.2 million metric tons of tomatoes annually and accounting for more than 36% of global production [2]. However, the cultivation of tomatoes is persistently impeded by many biotic and abiotic stresses, including soil infertility, pathogen invasion (bacteria, viruses, fungi, and nematodes), and environmental factors (salt, temperature, and drought) [3,4]. These stresses not only decrease crop yield but impair fruit quality, necessitating the implementation of sustainable agricultural approaches to improve tomato growth and yield [5,6]. Over the past few decades, the application of plant-growth-promoting (PGP) microbes (bacteria and fungi) has emerged as a viable environmentally friendly technique to increase crop resilience and productivity under these stresses [7,8]. Thus, it is suggested that by applying PGP microbes, we may reduce the use of chemical fertilizers and pesticides for healthier crop production in sustainable agriculture.

The plant-associated microbiota serves as a plant secondary genome [9], and the coevolution of these microbial communities with their host plants has led to complex symbiotic relationships [10]. In sustainable agriculture, the symbiotic relationship between plants and microbes is crucial for plant growth, productivity, development, and disease suppression [11]. Among the vast array of PGP microbes, bacterial endophytes are attracting much interest because of their ability to colonize internal plant tissues without causing harm [12,13]. They form symbiotic associations with their host plants and facilitate numerous physiological processes directly and indirectly [14]. The mechanisms include the synthesis of phytohormones, including indole-3-acetic acid (IAA), cytokinins, and gibberellins; nitrogen fixation; solubilization of vital elements such as phosphorus, potassium, and essential micronutrients; and the excretion of antimicrobial substances and volatile organic compounds that protect against phytopathogens [15,16,17]. Many previous studies have documented the role of bacterial endophytes as biofertilizers in enhancing plant health, growth, and performance [9,18]. Numerous PGP microbes have been reported to influence root architecture, increase shoot elongation, and elevate chlorophyll content and photosynthetic efficiency [19,20]. Therefore, we suggest that the use of bacterial endophytes as biological tools aligns with sustainable agricultural approaches, providing an environmentally favorable substitute for traditional agrochemicals.

Members of bacterial genera such as *Bacillus*, *Pseudomonas*, *Lysobacter*, and *Streptomyces* are well-known PGPs and are widely used in sustainable agriculture for disease control and plant growth promotion [8,21,22]. *Pseudomonas* species are motile, rod-shaped, Gram-negative bacteria that may grow under various ecological conditions. Numerous studies have demonstrated that *Pseudomonas* spp. are commonly used as PGPs and for disease suppression because of their different metabolic capacities [19,22]. For example, *P. chlororaphis* JF37 and *P. putida* WCS358 isolated from potato rhizosphere promoted plant growth in maize and *Arabidopsis thaliana* [23]. *P. koreensis* IB-4 significantly increased potato plant growth and yield by synthesizing cytokinin-like compounds and IAA and showed intense antifungal activity against *Alternaria*, *Bipolaris*, and *Fusarium* [24]. *P. aeruginosa* possesses genes for IAA and tryptophan biosynthesis, hence facilitating in promoting tomato plant growth [25]. Pham and colleagues reported that *P. stutzeri* A15, isolated from the rice rhizosphere, significantly enhanced rice seedling growth compared with untreated seedlings and can be used as a biofertilizer because of its nitrogen-fixing abilities [26]. Similarly, tomato plants inoculated with *P. koreensis* GS showed higher leaf chlorophyll contents, vigorous growth, and shoot biomass accumulation than control plants [22].

Over the past decade, advancements in high-throughput methodologies, including metabolomics, metagenomics, and transcriptomics, have made research on plant–microbe interactions easier, permitting thorough understanding of the biocontrol and PGP mechanisms of PGP microbes [18,27]. For instance, a study involving metagenomic and metabolomic analysis showed that pathogen-driven *Pseudomonas* mitigated *Fusarium* wilt disease caused by *F. oxysporum* in *Pseudostellaria heterophylla* by reshaping of the phyllosphere microbiome, biosynthesis of volatile organic compounds, and indirect antagonism of pathogen [28]. Similarly, by employing 16S amplicon sequencing, Dai and colleagues [8] reported that seed coat treatment with *Lysobacter antibioticus* 13-6 enhanced maize plant growth and yield by manipulating the host bacteriome and recruiting beneficial microbes. Comparative transcriptome analysis of tomato and potato plants treated with *P. fluorescens* SLU99 revealed that strain SLU99 promoted plant growth by inducing the expression of plant-hormone-related genes, including genes encoding gibberellic acid, cytokinin, and auxin [19]. Through transcriptome profiling, Guo and colleagues reported that the application of *P. koreensis* upregulated the expression of genes related to MAPK signal transduction, plant hormone biosynthesis, polyphenol biosynthesis, and stress tolerance in tomato plants [19]. In another study, Liu et al. [29] explored the PGP mechanism of *Paenibacillus polymyxa* YC0136 on tobacco through transcriptome profiling. They found that the application of *P. polymyxa* YC0136 promoted tobacco plant growth by inducing the expression of plant-hormone-related genes such as cytokinin, auxin, and gibberellin.

In our previous study, *Pseudomonas oryzihabitans* GDW1 isolated from the needles of healthy pine trees exhibited strong nematocidal activity against the pinewood nematode (PWN) *Bursaphelenchus xylophilus* and significantly mitigated the incidence of pine wilt disease (unpublished data). We observed that pine seedlings combined treated with PWN + GDW1 showed normal growth and lush green as compared with pine seedlings treated solely with PWN. Many previous studies have reported that a bacterium with strong potential as a biocontrol agent could be used as a plant growth promoter [8,30]. However, the PGP potential of *P. oryzihabitans* GDW1 for pine is unclear; thus, we used tomato as a model plant to investigate the potential of GDW1 as a PGP bacterium. Many previous studies have demonstrated the PGP potential of rhizospheric or endophytic *P. oryzihabitans* isolated from agricultural crops [31,32], and a limited number of studies have focused on endophytic bacteria isolated from perennial plants. In this study, we explored the underpinning PGP mechanism of *P. oryzihabitans* GDW1 on tomato plants isolated from a pine tree through a comprehensive multiomics approach, focusing on the modulation of the host transcriptome and bacteriome complexity. This approach allowed us to identify key genes, pathways, and microbial interactions involved in this beneficial symbiosis. We hypothesized that *P. oryzihabitans* GDW1 promotes tomato plant growth by increasing the expression of stress-tolerance- and plant-hormone-related genes and by reshaping the host root bacteriome. This study provides the first evidence of host bacteriome and transcriptome alterations in tomato plants treated with *P. oryzihabitans* GDW1 isolated from a perennial tree. Overall, our results provide evidence that endophytes from perennial trees and forests could be used as a new microbial source of PGP for sustainable agriculture to minimize the dependence on chemical fertilizers.

## 2. Results

### 2.1. Pseudomonas oryzihabitans GDW1 Enhances Tomato Plant Growth and Biomass Accumulation

**Figure 1:**
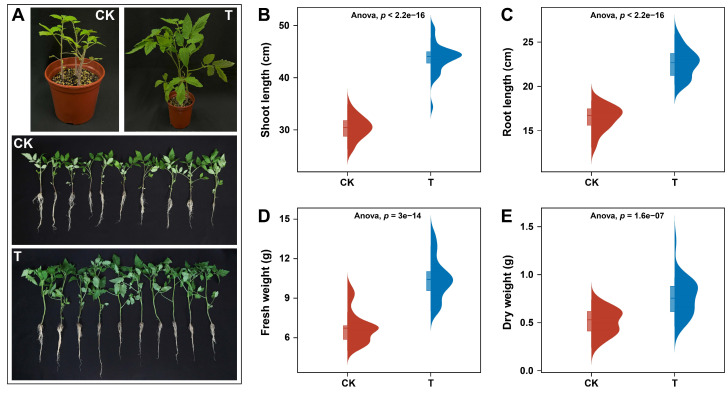
Effects of *Pseudomonas oryzihabitans* GDW1 on tomato plant growth and biomass accumulation. (**A**) differences in the growth of tomato plants treated with *P. oryzihabitans* GDW1 (T) and control (CK); (**B**) shoot length, (**C**) root length, (**D**) fresh weight, and (**E**) dry weight of tomato plants treated with *P. oryzihabitans* GDW1 (T) and control (CK). Significant differences among treatments are shown by univariate variance analysis of variance (ANOVA) at *p* < 0.05. T: application of *P. oryzihabitans* GDW1; CK: application of water as control.

The plant-growth-promoting (PGP) potential of *P. oryzihabitans* GDW1 was assessed in tomato plants in a pot experiment after one month of inoculation. Results of the greenhouse pot experiment showed that the application of GDW1 significantly enhanced the growth of tomato plants compared with untreated plants (Figure 1). An apparent difference was observed in the development of tomato plants treated with *P. oryzihabitans* GDW1 (T) as compared with control (CK) (Figure 1A). The results of univariate variance statistical analysis of plant growth indicators between different treatments revealed that tomato plant shoot length (cm), root length (cm), and biomass accumulation (fresh and dry weight (g)) were significantly higher under GDW1 treatment than in CK (ANOVA; *p* < 0.05, Figure 1A–D). The shoot length, root length, fresh weight, and dry weight of tomato plants treated with GDW1 were increased by 44%, 38%, 54%, and 59%, respectively, compared with control.

### 2.2. Transcriptome Profiling and Differentially Expressed Gene Analysis

**Figure 2:**
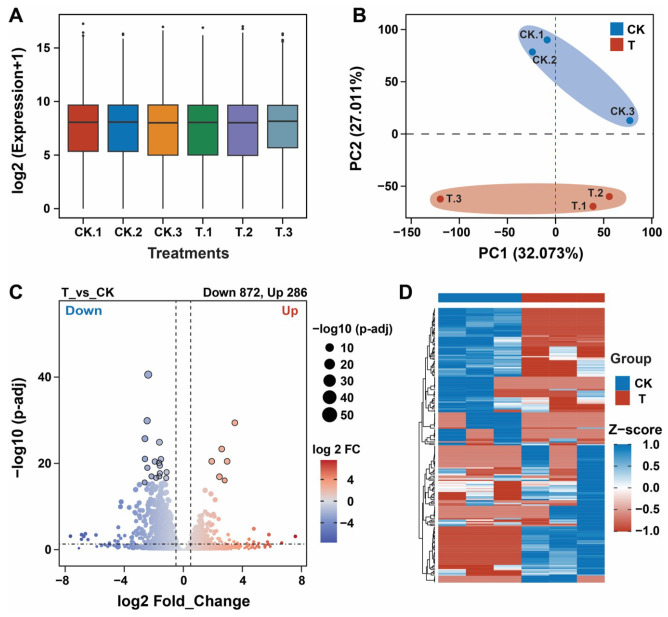
Transcriptome analysis of tomato roots treated with *Pseudomonas oryzihabitans* GDW1 compared with control. (**A**) Box plot showing the gene expression levels of all samples (CK.1, CK.2, CK.3, T.1, T.2, and T.3) under different treatments; (**B**) principal component analysis for samples (CK.1, CK.2, CK.3, T.1, T.2, and T.3) under different treatments; (**C**) volcano plot of DEGs (statistical significance at −log10 (*p*-adj; *y*-axis) and log2_FC (*x*-axis); and (**D**) heatmap of all DEGs in the groupwise comparison (T_vs_CK). T: application of *P. oryzihabitans* GDW1; CK: application of water as a control. CK.1, CK.2, CK.3, T.1, T.2, and T.3 are replicates per treatment, and each replicate contained the roots of five tomato plants.

Six tomato root samples from the control (CK) and treatment (T) groups were analyzed on an Illumina sequencing platform to confirm the changes in the transcriptomes of the tomato plants after the application of *P. oryzihabitans* GDW1. Results demonstrated that the application of GDW1 changed the transcriptome of the tomato plants compared with that of the control plants (Figure 2 and Table 1). A total of 304,740,186 raw reads were obtained from the six root samples. After quality control, 257,848,646 clean reads were obtained with a clean data ratio of 91.59%. The clean reads resulted in 251,346,481 mapped reads and a mapped alignment efficacy of 97.49% with the reference genome (*Solanum lycopersicum*). The reads had 98.6% and 94.69% Q20 and Q30 base (%) ratios, respectively, with GC content ratios between 44.12% and 44.58% (Table 1). The clean reads were first processed by DSEq2 v1.40. software for log2 normalization of gene expression levels. The results were visualized by box plots, which revealed that all six samples yielded the same expression level suitable for differentially expressed gene (DEG) analysis (Figure 2A). Principal component analysis revealed 59.084% variation in the transcripts of the treatment and control group (Figure 2B). Further DEG analysis was performed using the DSEq2 software at a threshold level of adj.*p*.value < 0.05 and |Log2FC| > 0.5. A total of 1158 DEGs were identified in the treatment (T) group compared with the CK group, including 286 upregulated and 872 downregulated genes (Figure 2C). The expression levels of all DEGs were visualized using a heatmap (Figure 2D).

### 2.3. Different Pathway Enrichment Analyses

**Figure 3:**
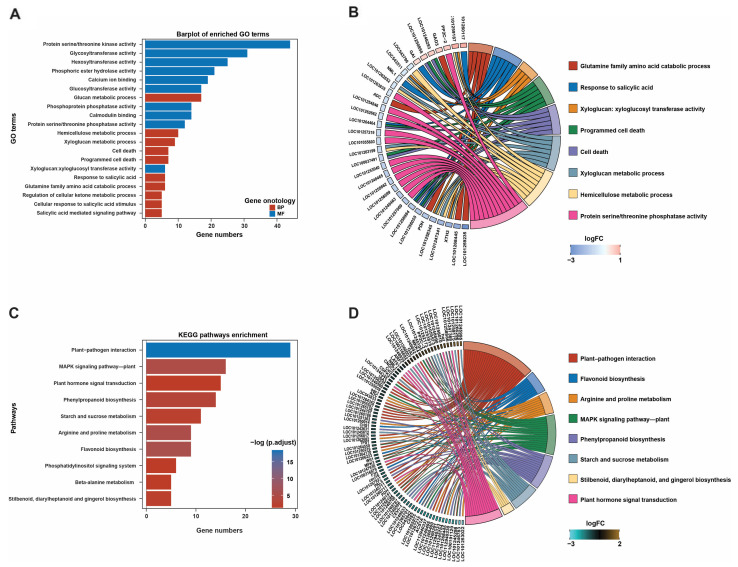
GO and KEGG pathway enrichment analyses of differentially expressed genes. (**A**) Bar plots of the top 20 enriched GO terms, (**B**) circos plot showing the annotation of DEGs in each GO term, (**C**) bar plots showing the top 10 enriched KEGG pathways, and (**D**) circos plot demonstrating the annotation of DEGs in each KEGG pathway.

We used the cluster_profile package in R to perform GO and KEGG enrichment analyses of all DEGs, and enriched pathways were screened under conditions of *p* < 0.05 and count > 1 (Figure 3). All 1158 DEGs were annotated into 156 classes of biological process (BP), cellular component (CC), and molecular function (MF) categories according to their GO terms (Table S1). Among the 156 classifications, the DEGs were significantly enriched in the top 20 GO terms, including protein serine/threonine kinase activity, glucan metabolic process, calcium ion binding, xyloglucan metabolism process, salicylic acid stimulation, and other pathways (Figure 3A). A circos plot shows the relationships between the top eight enriched GO pathways and their associated DEGs (Figure 3B). Furthermore, KEGG enrichment analysis was performed for the DEGs to gain insights into the biochemical pathways affected by the application of GDW1. It was found that the DEGs were significantly enriched in 10 KEGG pathways, such as plant–pathogen interaction (29 DEGs), MAPK signaling pathway—plant (16 DEGs), plant hormone signal transduction (15 DEGs), phenylpropanoid biosynthesis (14 DEGs), and others (Figure 3C and Table S2). A circos plot further visualizes the annotation of enriched DEGs in each KEGG pathway (Figure 3D).

### 2.4. Analysis of the Protein–Protein Interaction (PPI) Network and Hub Gene Screening

**Figure 4:**
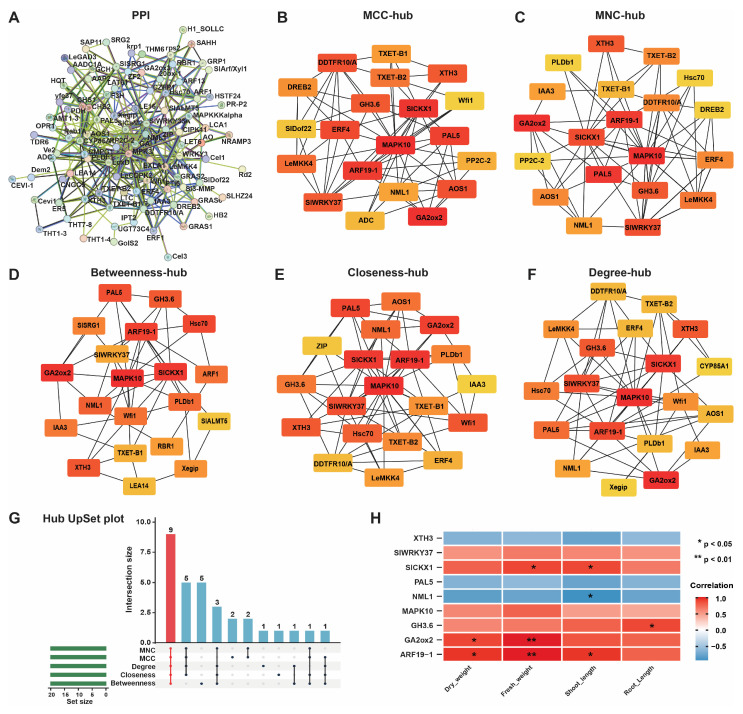
Protein–protein interaction (PPI) network and hub gene selection analyses. (**A**) PPI network analysis of differentially expressed genes; (**B**) MCC, (**C**) MNC, (**D**) betweenness, (**E**) closeness, and (**F**) degree for the identification of hub genes in the PPI network. (**G**): UpSet plot for hub genes and (**H**) heatmap for correlation analysis of nine hub genes and growth indicators. Nodes highlighted in red indicate greater measures of MNC, MCC, betweenness, closeness, and node degree within the network. Nodes colored red and yellow indicate medium MNC, MCC, betweenness, closeness, and node degree, while nodes colored yellow indicate lower MNC, MCC, betweenness, closeness, and node degrees in the network.

**Figure 5:**
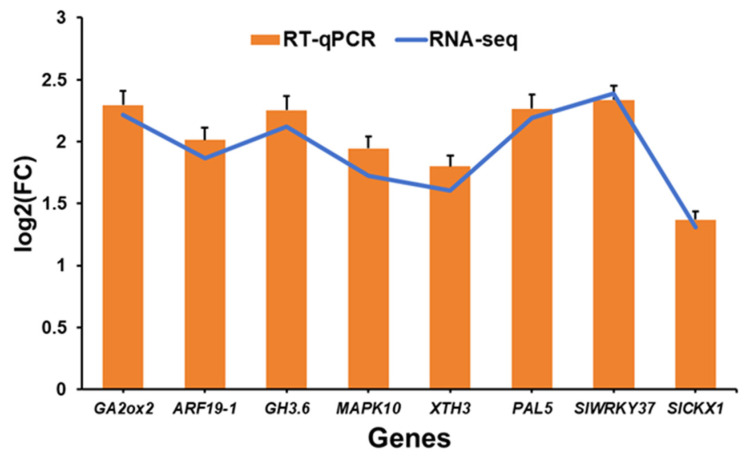
RT-qPCR assay for the validation of RNA-seq data.

The majority of proteins execute their biological functions via intermolecular interactions. The PPI network was constructed to gain insight into the biological functions of the DEGs and for the screening of hub genes, which play essential roles in metabolic activities (Figure 4). Proteins participating in the same metabolic pathway, biological process, structural complex, functional association, or physical contact act as nodes, and their interactions with proteins act as edges, in a PPI network. We used the STRING website to construct a PPI network for 1158 DEGs, with a confidence level of 0.2 and a species of *Solanum lycopersicum* resulting from a PPI containing 92 nodes and 176 edges (Figure 4A). Hub genes refer to genes that play important roles in the network; the loss of these genes would lead to the collapse of the entire network. To screen out hub genes in the PPI network, we used five algorithms: MCC (maximal clique centrality), MNC (maximum neighborhood component), betweenness, closeness, and degree in Cytoscape (v3.9.0) using the cytoHubba plugin. The top 20 genes in each algorithm with the highest MCC, MNC, betweenness, closeness, and degree scores were identified as hub genes (Figure 4B–F and Table S3), and the hub gene *MAPK10* was identified as the common gene in all algorithms. Furthermore, we generated a hub UpSet plot to select the genes that coexisted in the five algorithms as hub genes for subsequent analysis (Figure 4G). This analysis revealed nine genes (*XTH3*, *SlWRKY37*, *SlCKX1*, *PAL5*, *NML1*, *MAPK10*, *GH3.6*, *GA2ox2*, and *ARF19-1*) as hub genes that played important roles in the network as core genes. The expression of these hub genes was upregulated to varying degrees from log2FC 1.31~2.39 except for NML1, the expression of which was downregulated (log2FC = −0.55, Table S4). Additionally, according to Spearman correlation, a correlation analysis was performed between core genes and growth indicators. Genes related to PGP, including *SlCKX1*, *GH3.6*, *GA2ox2*, and *ARF19-1*, were significantly positively correlated to growth indicators. In contrast, other hub genes, including *SlWRKY37*, *PAL5*, and *MAPK10*, are known to be involved in plant defense and stress responses (Spearman, *p* < 0.05; Figure 4H). To validate the observed gene expression of hub genes obtained from the rRNA-seq data, we performed RT-qPCR on these hub genes, and the results of RT-qPCR were consistent with the rRNA-seq data, as the all-amplified genes showed increased gene expression (Figure 5).

### 2.5. Pseudomonas oryzihabitans GDW1 Influences the Assembly, Diversity, and Composition of Tomato Root Bacteriome

**Figure 6:**
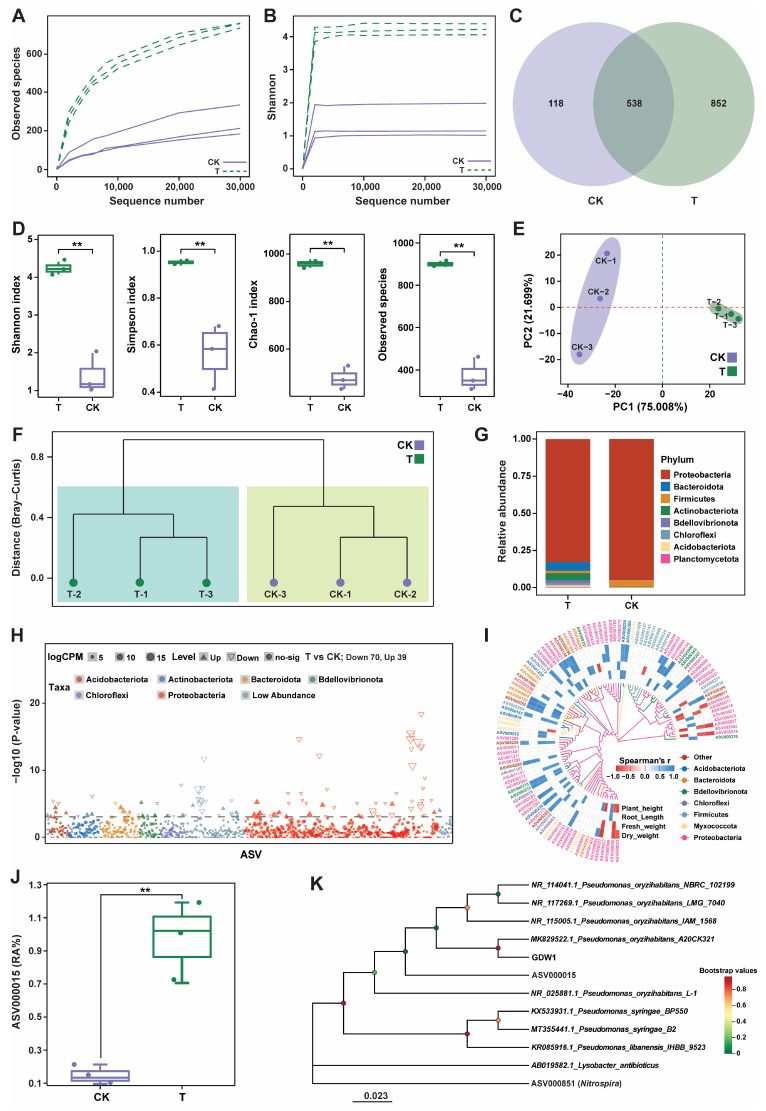
Impact of *Pseudomonas oryzihabitans* GDW1 on the diversity and composition of tomato root bacteriome. Observed species (**A**) and Shannon (**B**) index diversity rarefaction curves. (**C**) Venn diagram of amplicon sequence variants (ASVs). (**D**) Alpha diversity indices of root bacterial communities. (**E**) Principal component analysis based on the Euclidean distance matrix. (**F**) Hierarchical clustering analysis based on a Bray–Curtis distance matrix at the species level. (**G**): Bar plots for the relative abundance of the top 10 bacterial phyla. (**H**): Manhattan plot showing the differentially expressed ASVs. (**I**): Correlation analysis of plant growth parameters and differentially expressed ASVs at the phylum level. (**J**): Relative abundance (%) of ASV000015 in the treatment and control groups. (**K**): Phylogenetic tree showing the relationship between ASV000015 and *P. oryzihabitans* GDW1 according to the maximum likelihood method. Here, T: application of *P. oryzihabitans* GDW1; CK: application of water as control. ** *p* < 0.01 indicates a significant difference among treatments according to Student’s *t*-test.

The impact of *P. oryzihabitans* GDW1 on the tomato root bacteriome assembly, structure, and composition was studied by amplifying the V5–V7 variable region of the 16S rRNA gene (Figure 6). The 16S amplicon sequencing of six samples resulted in 753,818 raw reads. After quality control of the raw reads and chimera removal, a total of 490,510 effective tags (avg; 81,751 per sample) were obtained, with an effective tag ratio of 65.06%. These effective tags were further used for taxonomic annotation to obtain amplicon sequence variants (ASVs), which resulted in a total of 3822 ASVs with an average of 637 ASVs per sample. The statistics of the raw data collected from 16S amplicon sequencing of the host bacteriome are shown in Table S5. First, we generated rarefaction curves of all samples to verify whether the sequencing data adequately reflected the diversity and abundance of the species in the samples. As the sequencing depth increased, the curve tended to be flattened, indicating that each sample sequence was sufficient to reflect the species diversity and that the amount of sequencing data met the needs of the analysis (Figure 6A,B). The taxonomic annotation of the ASVs revealed 538 core ASVs in different groups: 852 were unique to the T group, and 118 were unique to the control group (Figure 6C). Alpha diversity analysis demonstrated that strain GDW1 could affect the Shannon, Simpson, Chao-1, and observed species indices of bacterial communities. The values of these indices were found to be significantly higher in the treatment (T) than the control (CK) group (Wilcoxon-test, *p* < 0.01; Figure 6D). Principal component analysis (PCA) based on the Euclidean distance matrix showed apparent differences between the CK and T samples. The first two axes (PCA-1 and PCA-2) of PCA demonstrated 96.707% (PERMANOVA, *p* = 0.001 and R^2^ = 0.8259) of the total variation in the structure of the bacterial communities under different treatments (Figure 6E). Further hierarchical clustering analysis based on a Bray–Curtis distance matrix at the species level revealed that application of strain GDW1 altered the bacterial communities in T than CK, as CK and T samples were divided into two clades (Figure 6F).

Bar plots at the phylum level revealed that the application of GDW1 significantly altered the community composition compared with the control. The relative abundance results of species at the phylum level revealed that Proteobacteria was the dominant phylum in both CK and T. However, the relative abundances of Bacteroidota, Actinobacteriota, Bdellovibrionota, and Chloroflexi increased in the treatment group, and the abundance of Firmicutes was higher in the control group (Figure 6G). We further used the edgeR package to perform variance analysis between the T and CK groups at a threshold level of *p* < 0.05 and |Log2FC| > 1 to screen the keystone taxa. There were 109 significantly differentially expressed ASVs in T compared with those in CK, including 39 upregulated and 70 downregulated ASVs (Figure 6H). The Manhattan plot results revealed that Proteobacteria was the dominant phylum with the maximum number of differential ASVs, followed by Acidobacteriota and Bacteroidota. We then extracted 109 differentially expressed ASVs and performed a correlation analysis with growth indicators. The results showed that 53 differential ASVs were significantly correlated with growth indicators, with Proteobacteria being the main responsive taxon. ASV000015 showed a significant positive correlation with growth indices (Figure 6I). The abundance of ASV000015, affiliated with the phylum Proteobacteria, was significantly increased in the T group compared with the CK group (Figure 6J). Furthermore, the phylogenetic tree constructed using the maximum likelihood method showed that ASV000015 was present in the same clade as *P. oryzihabitans* and was closely related to *P. oryzihabitans* GDW1 (Figure 6K). However, the source of the microbes that constituted the new microbiome in tomato roots treated with *P. oryzihabitans* GDW1 was likely multifaceted. Hence, it is assumed that these microbes might have been the plant’s endogenous endophytic microbiome. Additionally, the endophytic nature of GDW1 suggests that it may facilitate the recruitment of microbes from the surrounding environment, thereby reshaping the root bacteriome. Therefore, more in-depth studies are required to understand the origin of these microbes to optimize the application of GDW1 in agricultural practices.

### 2.6. Application of Pseudomonas oryzihabitans GDW1 Increases Bacterial Network Complexity

**Figure 7:**
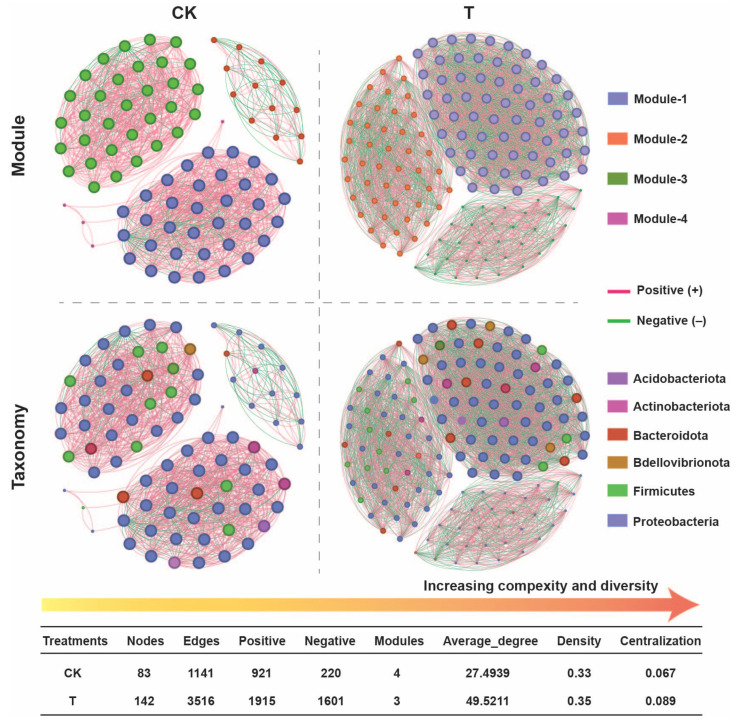
Impact of *Pseudomonas oryzihabitans* GDW1 on tomato roots’ endophytic bacterial ecological network complexity. The tomato root bacterial co-occurrence network was constructed using the ASV table. The red lines in the networks indicate strong positive correlations, whereas green lines signify negative correlations, according to Spearman’s correlation at *p* < 0.05. The topological metrics of the co-occurrence network of each treatment are listed in the table. T: application of *P. oryzihabitans* GDW1; CK: application of water as a control.

To investigate the impact of biological interactions on the coexistence of root-associated bacteriomes, we constructed a bacterial co-occurrence network of *P. oryzihabitans* GDW1 treated and control groups by eliminating the ASVs with relative abundances less than 0.005 (Figure 7). The results suggested that the bacterial network complexity significantly increased after *P. oryzihabitans* GDW1 (T) application compared with CK. The network of the treatment (T) group was divided into three modules, which showed a complex bacterial network with a greater network complexity (average degree, 49.52) than that of CK (modules, 4; average degree, 27.49). These findings indicate that *P. oryzihabitans* GDW1 reduces resource competition between microbes and that internal members are relatively close. Network density and centralization increased under the *P. oryzihabitans* GDW1 treatment (0.35 and 0.089, respectively) compared with the control (0.33 and 0.067, respectively). Notably, the numbers of nodes (143) and edges (3516) were significantly higher under *P. oryzihabitans* GDW1 treatment than in the control (nodes, 83 and edges, 1141). The application of *P. oryzihabitans* GDW1 significantly increased the positive interaction among the microbes (positive nodes = 1915) compared with CK (positive nodes = 921). Further network analysis at the taxonomic level exhibited a consistent pattern with a modular network, and most nodes represented Proteobacteria (41% to 48%) and Bacteroidota (5% to 6%).

### 2.7. Functional Prediction Analysis

**Figure 8:**
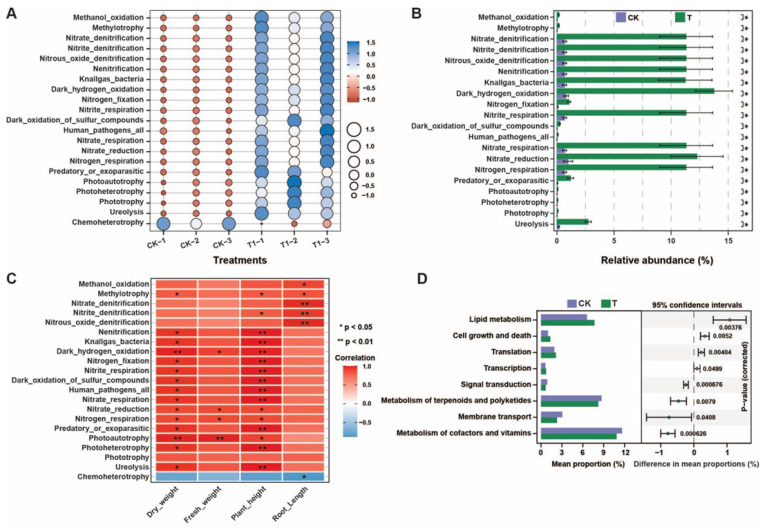
Differences in the functional prediction analysis of root bacterial communities under different treatments. (**A**) Bubble chart of the FAPROTAX results, (**B**) FAPROTAX difference analysis results and * *p* < 0.05 indicates a significant difference among enriched pathways according to Student’s *t*-test, (**C**) correlation analysis between FAPROTAX difference results and plant growth indicators, and (**D**) PICRUSt2 KEGG level 2 prediction difference analysis of bacterial communities. T: application of *P. oryzihabitans* GDW1; CK: application of water as control.

We used FAPROTAX (v.1.2.1.0) and the PICRUSt2 (v.2.5.0) tools for the functional prediction of microbial communities regulated by the application of *P. oryzihabitans* GDW1 (Figure 8). Results of FAPROTAX analysis demonstrated that pathways such as nitrogen respiration, ureolysis, nitrate reduction, nitrite respiration, nitrogen fixation, nitrite denitrification, methanol oxidation, and others, except for chemoheterotrophy, were significantly enriched in treatment compared with control (Figure 8A and Table S6). A significant difference was observed in the relative abundance of these enriched pathways according to Student’s *t*-test (*p* < 0.05, Figure 8B). The results of the correlation analysis with growth indicators showed that the pathways of ureolysis, nitrogen respiration, nitrate respiration, nitrogen fixation, nitrate denitrification, methanol oxidation, and others were positively correlated with growth indicators. In contrast, chemoheterotrophy was negatively correlated with growth indicators (Figure 8C). PICRUSt2 functional prediction of bacterial communities at KEGG level 2 revealed that transcription, translation, cell growth and death, and lipid metabolism were significantly higher in the treatment group than in the control group. In comparison, the metabolism of cofactors and vitamins, membrane transport, signal transduction, and metabolism of terpenoids and polyketides in the control group were higher than the treatment group (Figure 8D and Table S7).

### 2.8. Integration Analysis of Bacteriome and Transcriptome Data

**Figure 9:**
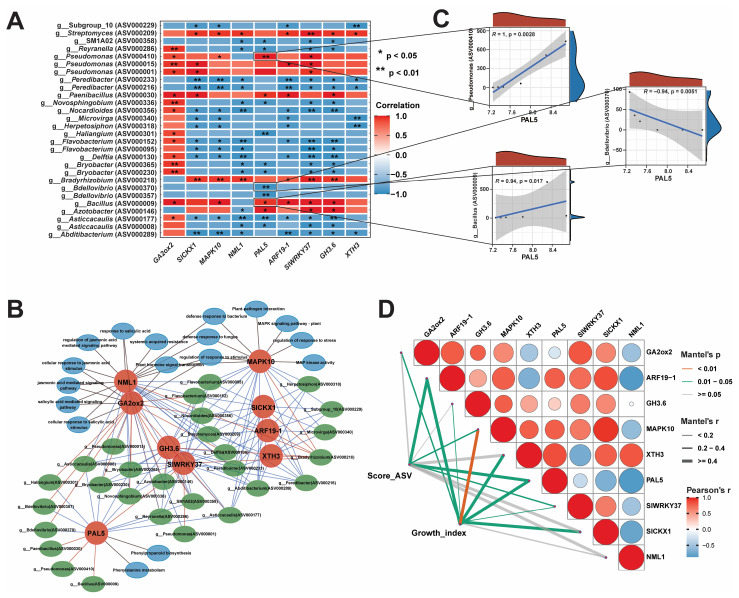
Correlation network analysis of the hub genes and ASVs. (**A**) Heatmap of correlations between key ASVs and hub genes, (**B**) ASV score–hub gene–regulatory pathway network analysis, (**C**) scatter plot of correlation between key ASVs and PAL hub genes, and (**D**) Score_ASV-Hub_gene- growth_growth index Mantel.test analysis.

Bacterial endophytes can regulate the expression of genes, which are involved in the development of host resistance to biotic and abiotic stresses. In order to explore the changes in the host, we performed GO and KEGG enrichment analyses on the hub genes to identify the pathways affected by changes in the microbial community (*p* < 0.05, Figure 9). The results revealed that DEGs were involved in several key pathways, such as salicylic-acid-mediated signaling, jasmonate-mediated signaling, phenylalanine metabolism, and phenylpropanoid biosynthesis. Additionally, DEGs were associated with the response to salicylic acid, regulation of stimulation response, response to bacterial defense, salt stress, osmotic stress, and cell response to organic cyclic compounds, jasmonate stimulation, and fatty acids. Subsequently, to analyze the regulatory relationship between the transcriptome and bacteriome, we conducted a joint analysis of nine hub genes and 53 ASVs related to growth indicators. The relationships between hub genes and key ASVs were analyzed using threshold level (*p* < 0.05, |R| > 0.8), and it was found that 28 ASVs had strong correlations with nine hub genes (Figure 9A). We further conducted a Score_ASV-Hub_gene pathway network analysis, and the resulting network was comprised of 54 nodes and 154 edges (Figure 9B). Score_ASV-Hub_gene pathway network analysis showed that the *PAL5* gene involved in the phenylalanine-related biosynthesis pathway showed significant positive correlations with g__*Bacillus* (ASV000009), g__*Azotobacter* (ASV000146), g__*Pseudomonas* (ASV000410), and g__*Paenibacillus* (ASV000030). Conversely, it exhibited significant negative correlations with g__*Novosphingobium* (ASV000336), g__*Reyranella* (ASV000286), g__SM1A02 (ASV000358), g__*Haliangium* (ASV000301), g__*Bdellovibrio* (ASV000370, ASV000375), and g__*Asticcacaulis* (ASV000177). We further used a scatter plot of the correlations between some ASVs and the *PAL5* gene, which indicated that these ASVs could regulate and affect the expression of the *PAL5* gene, affect the phenylalanine-related biosynthesis pathway, and stimulate systemic immune resistance (Figure 9C). The *GA2ox2* gene, which is involved in the gibberellin (GA) biosynthesis pathway, was strongly positively correlated with most of the AVSs, including g__*Pseudomonas* (ASV000410, ASV000015), g__*Bacillus* (ASV000009), and g__*Bryobacter* (ASV000365, ASV000230).

The *MAPK10* gene involved in the MAPK signaling pathway and pathogen-interaction-related pathways exhibited positive correlations with g__*Bradyrhizobium* (ASV000218), g__*Bacillus* (ASV000009), g__*Pseudomonas* (ASV000410), and g__*Streptomyces* (ASV000209) but had negative correlations with g__*Abditibacterium* (ASV000289), g__*Asticcacaulis* (ASV000177), g__*Delftia* (ASV000130), g__*Flavobacterium* (ASV000152), g__*Herpetosiphon* (ASV000318), g__*Nocardioides* (ASV000356), and g__*Peredibacter* (ASV000233). Some other genes with PGP properties, such as *SlCKx1*, *ARF19-1*, *GH3.6*, and *XTH3*, showed positive correlations with g__*Azotobacter* (ASV000146), g__*Bacillus* (ASV000009), g__*Bradyrhizobium* (ASV000218), g__*Paenibacillus* (ASV000030), g__*Pseudomonas* (ASV000410, ASV000015, ASV000001), and g__*Streptomyces* (ASV000209) (Figure 9A). Additionally, Mantel.test results revealed that 28 Score_ASVs were positively correlated with *ARF19-1* (r = 0.607, Mantel.test.*p* = 0.039), *MAPK10* (r = 0.289, Mantel.test.*p* = 0.002), *PAL5* (r = 0.614, Mantel.test.*p* = 0.048), and *SlWRKY37* (r = 0.253, Mantel.test.*p* = 0.043; Figure 9D). All hub genes, including *GA2ox2*, *ARF19-1*, *GH3.6*, *MAPK10*, *XTH3*, *PAL5*, *SlWRKY37*, and *SlCKX1*, exhibited positive correlation with growth_indexes (Table S8), except for *NML1* (r = 0.292, Mantel.test.*p* = 0.082; Table S8). These results indicate that the application of *P. oryzihabitans* GDW1 modulates the host bacteriome and regulates the expression of the hub genes *GA2ox2*, *ARF19-1*, *GH3.6*, *MAPK10*, *XTH3*, *PAL5*, *SlWRKY37*, and *SlCKX1*, which play important roles in improving tomato plant growth.

## 3. Discussion

In this study, we investigated the plant-growth-promoting (PGP) potential and underlying PGP mechanism of an endophytic bacterium, *Pseudomonas oryzihabitans* GDW1, isolated from the needles of a healthy pine tree. We used integrated multiomics techniques, such as host bacteriome analysis using 16S amplicon sequencing and transcriptomics profiling, to investigate the relationships between *P. oryzihabitans* GDW1 and tomato plants. These findings provide new insights into how *P. oryzihabitans* GDW1 facilitates tomato plant growth and indicate its potential application in sustainable agriculture. The result from the greenhouse pot experiment indicated that inoculation with *P. oryzihabitans* GDW1 markedly improved the growth of tomato plants, as shown by the increased lengths of shoots and roots, along with greater fresh and dry weights, compared with control plants. These results align with prior research emphasizing the growth-enhancing properties of *Pseudomonas* species [3,22,33]. The notable increase in plant biomass (up to 59% for dry weight) suggests that *P. oryzihabitans* GDW1 is an effective PGP bacterium that likely functions by increasing nutrient absorption and modulating plant metabolic pathways. Multiple mechanisms have been proposed for the PGP activity of *Pseudomonas* sp., including hormone synthesis, nutrient solubilization, and disease suppression [34,35]. The transcriptome and functional investigations of *P. oryzihabitans* GDW1 indicate the involvement of numerous pathways, including those involved in phytohormone production and stress tolerance. This complex mechanism of action highlights the ability of GDW1 to increase crop resilience against both biotic and abiotic challenges.

Transcriptome analysis identified 1158 differentially expressed genes (DEGs) in tomato plants inoculated with *P. oryzihabitans* GDW1. The expression of numerous essential genes involved in phytohormone signaling, especially those related to auxin, gibberellin, and cytokinin production, was markedly elevated. These hormones are necessary for plant growth, particularly in facilitating cell elongation and division, plant vigor, and root and shoot growth [36,37]. The activation of these pathways indicates that *P. oryzihabitans* GDW1 promotes growth by regulating the hormonal equilibrium of plants. In addition to hormone-related genes, DEGs associated with stress response pathways were also enriched. In *P. oryzihabitans* GDW1-treated tomato plants, genes linked to defense responses, plant response to biotic and abiotic stresses, and secondary metabolite production were elevated. These findings indicate that the bacterium not only facilitates growth but increases the plant’s capacity to withstand environmental stresses, a characteristic that may be especially beneficial in agricultural systems with escalating environmental challenges such as biotic and abiotic stresses [19,25]. KEGG pathway enrichment underscored the activation of pathways related to plant–pathogen interactions, secondary metabolite production, plant hormone signal transduction, and MAPK signaling, which are recognized for their roles in growth promotion and defense mechanisms [38,39].

The PPI network constructed for the DEGs had 92 nodes and 176 edges, indicating that proteins interacted to facilitate diverse biological processes. Through the use of five algorithms, MCC, MNC, betweenness, closeness, and degree, we discovered nine hub genes (*MAPK10*, *ARF19-1*, *SlCKX1*, *GA2ox2*, *PAL5*, *SlWRKY37*, *GH3.6*, *XTH3*, and *NML1*) within the PPI network. These genes participate in essential signaling networks associated with stress tolerance, hormone control, growth promotion, and plant defense systems [40,41,42]. In our study, transcriptome analysis of tomato roots treated with *P. oryzihabitans* GDW1 demonstrated that strain GDW1 could increase auxin, cytokinin, and gibberellin signal transduction in tomatoes. GA is necessary for tomato seed germination, leaf growth, and root elongation, and GA homeostasis is essential for normal plant growth and development [19,43]. *GA2ox* is a crucial enzyme that performs a negative regulatory function in the GA biosynthesis pathway, and the upregulation of *GA2ox* expression can reduce gibberellin levels and optimize plant structure [44]. Moreover, many previous studies have revealed that elevated expression levels of genes producing *GA2ox* aid plants in adapting to unfavorable environments, including drought and salt stresses [45,46]. *GA2ox2* encodes a gibberellin 2-oxidase, which catalyzes the deactivation of bioactive gibberellins (GAs). GAs are essential hormones that promote stem elongation, seed germination, and flowering. By reducing bioactive GA levels, *GA2ox2* prevents excessive GA signaling, ensuring proper development [47]. These findings suggest that the application of *P. oryzihabitans* GDW1 increases the expression of the *GA2ox2* gene in tomato roots, indicating that *P. oryzihabitans* GDW1 may help in maintaining the GA level for normal growth and development of tomato plants.

Studies have demonstrated that numerous PGP bacteria govern the localization and distribution of auxin within plants, which is essential for different morphological changes [48]. Treatment with PGP bacteria led to elevated endogenous auxin levels and was correlated with root development. However, the impact of exogenous auxin on root development was contingent upon its concentration, and root growth was inhibited at high concentrations of exogenous auxin [49]. In summary, whereas auxin significantly contributes to root growth, regulating endogenous auxin levels is equally essential. Our results demonstrated that the expression of the *GH3.6*, *SlCKX1*, and *ARF19-1* genes was elevated in the roots of tomatoes treated with *P. oryzihabitans* GDW1. *GH3.6* facilitates the conjugation of indole-3-acetic acid (IAA) with amino acids, resulting in the formation of IAA-amino acid conjugates [50]. By conjugating auxin, *GH3.6* can reduce auxin signaling, preventing overstimulation and maintaining a balanced auxin response [47]. The *ARF19* gene is essential for modulating plant responses to auxin, a significant hormone implicated in numerous developmental processes [51]. Studies have shown that *ARF7* and *ARF19* promote the auxin response and drive several aspects of plant development, such as leaf cell expansion and lateral root formation [51,52]. *GH3.6* plays a role in regulating hormonal equilibrium by transforming surplus-free IAA into inactive conjugates, thus ensuring auxin homeostasis [53]. *ARF19* interacts with Aux/IAA proteins, auxin-signaling repressors, to maintain the equilibrium between Aux/IAAs and ARFs, essential for optimizing plant responses to auxin [54]. The coordinated action of these hub genes maintains a delicate balance in hormone signaling. Increased *ARF19-1* expression amplifies auxin responses when needed, while *GH3.6* and *GA2ox2* act as negative regulators, preventing overstimulation by auxin and GAs, respectively [47,51,52]. This precise control over hormone levels is essential for various aspects of plant growth, including cell division, elongation, and differentiation [47,51]. Thus, we assumed that by preventing excessive or insufficient hormonal signaling, these hub genes contribute to optimized plant architecture, enhanced stress tolerance, and improved overall biomass accumulation.

Similarly, *SlCKX1* encodes an enzyme that catabolizes cytokinins, which are implicated in numerous developmental processes, including cellular division and shoot and root growth. Higher expression levels of *SlCKX1* regulate cytokinin levels by enhancing the degradation of cytokinins, hence sustaining hormonal equilibrium inside the plant for proper plant growth [55]. In addition to auxins, *GH3.6* is involved in regulating other phytohormones (SA and JA), demonstrating its role in intricate hormonal interactions necessary for plant adaptation [53]. The *SlCKX1* gene contains many cis-acting elements that respond to methyl jasmonate, auxin, and abscisic acid (ABA), signifying its participation in multiple signaling pathways associated with plant stress responses and growth regulation [55]. Thus, we assumed that the application of *P. oryzihabitans* GDW1 maintained plant hormone homeostasis (auxin and cytokinin), essential for normal plant growth and development, by increasing the expression of *ARF19-1*, *SlCKX1*, *GA2ox2*, and *GH3.6*. This finding is in accordance with the study of Hanifah and colleagues, who reported that the application of PGP *P. fluorescens* SLU99 induced the expression of hormone-related genes, specifically those responsible for regulating the homeostasis of gibberellic acid, auxin, ethylene, and cytokinin in tomato and potato [19]. Furthermore, relatively high expression levels of *ARF19-1*, *SlCKX1*, *GA2ox2*, and *GH3.6* in tomato roots strongly correlated with plant growth metrics, underscoring their ability to facilitate plant growth and resilience to environmental stresses via hormonal mechanisms.

The expression of hub genes such as *PAL5*, *MAPK10*, and *SlWRKY37* was upregulated in the transcriptome of tomato roots treated with *P. oryzihabitans* GDW1. *PAL5* encodes an enzyme that facilitates the transformation of phenylalanine into cinnamic acid, the initial step in the phenylpropanoid biosynthesis pathway. Plants produce phenylpropanoids as secondary metabolites from phenylalanine, which can improve plant resistance to diverse biotic and abiotic stresses [29]. The synthesis of phenolic compounds by PAL enzymes enhances plant defense mechanisms by fortifying cell walls and synthesizing antibacterial compounds [56]. *MAPKs* and *WRKYs* are proteins that influence the activity of transcription factors in plants and play important roles in plant signal transduction [57], plant growth and development [58,59], and plant tolerance to biotic and abiotic stresses [59,60]. Studies have revealed that *MAPK10* interacts with plant hormone ABA and that ethylene increases the plant’s ability to respond to biotic stresses and enhances plant growth [61]. The activation of *MAPKs* is associated with the overexpression of *WRKY* transcription factors, which are essential for controlling gene expression during pathogen invasion. The upregulation of hub genes related to phenylpropanoid biosynthesis, stress signaling, and plant signal transduction, such as *PAL5*, *MAPK10*, and *SlWRKY37*, further substantiates the concept that *P. oryzihabitans* GDW1 augments the robustness of tomato plants to stresses, including biotic and abiotic stresses, alongside PGP. Our study corresponds with earlier reports on tobacco and tomato that the application of PGP *P. polymyxa* YC0136 and *P. koreensis* enhanced plant growth by increasing the expression of plant-hormone-related genes such as auxin, cytokinin, and gibberellin, along with genes involved in the phenylpropanoid biosynthesis pathway, MAPK signaling transduction, and transcription factors associated with stress tolerance, specifically *WRKY* and *MYB* [22,29]. However, future research will enhance our understanding of the involvement of *P. oryzihabitans* GDW1 in regulating plant defense under biotic and abiotic stresses.

The investigation of the tomato root bacteriome demonstrated that inoculation with *P. oryzihabitans* GDW1 markedly modified the composition and diversity of the microbial community. The bacterial diversity indices (Shannon, Simpson, and Chao-1) were elevated significantly in the treated group, suggesting that *P. oryzihabitans* GDW1 promotes a more diverse and intricate root microbiome. Principal component analysis revealed distinct differences in the bacteriomes of treated and control plants, with significant microbial taxa, including Proteobacteria and Bacteroidota, exhibiting greater abundance in the treated plants. Prior research has indicated that PGP bacteria modify the host microbiome of host plants, increasing the prevalence of beneficial microorganisms while diminishing the presence of pathogens [27,28]. In our investigation, *P. oryzihabitans* GDW1 increased the abundance of taxa associated with nitrogen cycling and organic matter decomposition, indicating increased nutrient availability for the host plant. The co-occurrence network analysis demonstrated a more intricate and interconnected microbial network in the *P. oryzihabitans* GDW1-treated plants. These findings suggest that bacteria promote advantageous microbial interactions that likely increase plant development and health. These results are similar to the study of Dai and colleagues, who reported that the application of *L. antibioticus* 13-6 enhanced the maize plant rhizosphere network complexity by reducing the competition for resources and increasing the abundance of beneficial microbial taxa [8]. A differential expression study of amplicon sequence variations (ASVs) revealed 109 significantly changed ASVs in treated plants, with numerous ASVs, especially those from the phylum Proteobacteria, exhibiting strong relationships with plant growth traits. These findings indicate the beneficial effects of *P. oryzihabitans* GDW1 may, in part, be facilitated by its impact on the host microbiota. These results are in accordance with the findings of Tao and colleagues, who reported that bio-organic fertilizers (containing *Bacillus amyloliquefaciens* W19) promoted plant growth and enhanced disease resistance by stimulating indigenous soil *Pseudomonas* populations [62]. Moreover, through ASV differential analysis, we found that ASV000015, which belongs to the phylum Proteobacteria, was more abundant in the roots of tomato plants treated with *P. oryzihabitans* GDW1 than in control plants. Many previous studies have demonstrated that members of Proteobacteria promote plant growth under biotic and abiotic stresses by producing specific secondary metabolites, indole acetic acid (IAA), and siderophores and solubilizing phosphates [8,63]. It is important to note that the findings presented in this study are based on a specific model setup involving tomato plants grown under controlled greenhouse conditions, and the results may vary under different conditions. While these results provide valuable insights into the PGP mechanisms of *P. oryzihabitans* GDW1, the composition and dynamics of the microbiome may vary under various environmental conditions, soil types, or host plants. Therefore, long-term studies are needed to understand the impact of *P. oryzihabitans* GDW1 on the temporal dynamics of the host bacteriome and plant growth and health across different ecosystems.

Furthermore, functional prediction analysis of microbial communities associated with tomato plants inoculated with *P. oryzihabitans* GDW1 was performed using FAPROTAX and PICRUSt2. FAPROTAX analysis revealed that the dominant bacterial communities were involved in nitrogen cycling, organic matter degradation, and plant growth promotion, indicating a beneficial role of *P. oryzihabitans* GDW1 in enhancing soil health and nutrient availability. Meanwhile, PICRUSt2 predictions suggested significant enrichment of pathways related to plant hormone signaling and stress response, which aligned with the improvements in tomato growth metrics and resilience against abiotic stresses. These results are in accordance with previous studies showing that the genome of *P. oryzihabitans* CB24 harbors genes responsible for nitrogen acquisition, iron acquisition, sulfur assimilation, nitrogen acquisition, and phytohormone production. Thus, it showed the potential of *P. oryzihabitans* CB24 to stimulate plant growth and alleviate biotic and abiotic stresses [33]. These results suggest that *P. oryzihabitans* GDW1 has the potential to be used as a biofertilizer in sustainable agriculture.

Additionally, integrated host bacteriome and transcriptomic analysis elucidated the mechanisms by which *P. oryzihabitans* GDW1 enhances plant growth through many pathways. A correlation study of hub genes and important ASVs demonstrated significant connections between microbial taxa and gene expression patterns associated with growth and defense. The *PAL5* gene, the main gene in the regulatory network involved in secondary metabolite biosynthesis, was positively correlated with ASVs from the genera *Bacillus*, *Azotobacter*, and *Pseudomonas*, suggesting that these bacteria may affect the expression of plant-defense-related genes. Our results are consistent with previous studies that the application of *P. polymyxa* YC0136 upregulated the expression of genes involved in phenylpropanoid biosynthesis by 1.51–4.59 times [29]. Similarly, Durairaj and fellows reported that *P. aeruginosa* and *B. stratosphericus* enhanced tomato plant resistance to various bacterial pathogens, including *Burkholderia glumae*, *Xanthomonas oxyzae* pv. *Oryzae*, *P. syringae* (KACC 15 105), *Pectobacterium carotovorum*, and *Ralstonia solanacearum*, by triggering the expression of defense-related genes (*PR-1a* and *PAL*) [64]. This regulatory network study further emphasized the role of key ASVs in regulating growth and stress response pathways. The interaction between plant gene expression and the host bacteriome indicates that *P. oryzihabitans* GDW1 directly influences plant metabolic processes and improves plant health by altering the host bacterial population.

## 4. Materials and Methods

### 4.1. Bacterial Strain and Growth Conditions

*Pseudomonas oryzihabitans* GDW1, an endophytic strain with direct nematicidal activity, was previously isolated from the needles of a healthy pine tree. The strain was stored in 50% glycerol solution (*v*/*v*) at −80 °C in our laboratory, the Guangdong Province Key Laboratory of Microbial Signals and Disease Control, College of Plant Protection, South China Agricultural University, China. *P. oryzihabitans* GDW1 was reactivated on Luria–Bertani (LB) agar media (10 g/L bacto tryptone, 5 g/L yeast extract, 10 g/L NaCl, 18 g/L agar, and pH 7.0) and cultured overnight at 28 °C [27]. A single colony was picked from the pure culture, transferred into 50 mL of LB broth, and incubated at 28 °C and 180 rpm/min for 12 h to prepare the seed solution. Then, 10 mL of seed solution was transferred into 100 mL of fresh LB broth and cultured overnight at 28 °C and 180 rpm/min. The cell density of the obtained culture medium was adjusted to an OD_600 nm_ = 0.6 (~10^8^ CFU/mL) using a spectrophotometer (GE Uitrospec 2100 pro; GE Healthcare, Tokyo, Japan) for the plant growth promotion assay in the pots.

### 4.2. Plant Material and Nursery Raising

Seeds of the tomato cultivar “Xin Jin Feng No.1” were obtained from Guangzhou Changhe Seed Co., Ltd. (Guangzhou, China) and used as plant material. Tomato seeds were first soaked in sterilized distilled water (sdH_2_O) for 12 h to remove floating seeds and impurities. The seeds were then surface sterilized with 10% NaClO for 2 min and 75% ethanol for 30 s, followed by three times washing with sdH_2_O to generate disease-free seedlings. The sterilized tomato seeds were sown and germinated in standardized plug trays (540 mm × 280 mm) containing sterilized peat, vermiculite, and perlite (3*V*:*V*:*V*) as substrates and placed in a growth chamber for 2 weeks under a controlled environment with a relative humidity of 75% and temperature 25 ± 1 °C [65].

### 4.3. Greenhouse Pot Experiment

The greenhouse pot experiment was conducted in the greenhouse of the College of Plant Protection, South China Agricultural University, Guangzhou (23.1291° N, 113.2644° E), China. Tomato seedlings at two true leaf stages were grown in flower pots filled with sterilized (autoclaved) soil (peat–vermiculite–perlite = 2:1:1), with three seedlings in each pot and placed in the greenhouse under controlled environmental conditions as follows: relative humidity of 75% and day (25 ± 1 °C) /night (20 ± 1 °C) temperature [66]. After 5 days of acclimatization, the pots with uniform heights were selected to conduct the experiment. The experiment was performed under two conditions: CK, seedlings inoculated with 30 mL/pot of sdH_2_O as control, and T, seedlings inoculated with 30 mL/pot of *P. oryzihabitans* GDW1 (~10^8^ CFU/mL) LB suspension. The experiment was performed in a completely randomized block design and was repeated thrice, with five pots in each treatment as replicates.

### 4.4. Assessment of Plant-Growth-Promoting Traits

The plant-growth-promoting potential of *P. oryzihabitans* GDW1 on tomato plants was assessed by determining shoot length, root length, fresh weight, and dry weight. One month after inoculation, ten plants from each treatment were uprooted and gently washed under tap water to remove any soil and debris, and shoot length (cm), root length (cm), fresh weight (g), and dry weight (g) were recorded as described by Liu [29]. These plants were dried in an oven at 80 °C to a constant weight for 6 h for dry weight assessment.

### 4.5. Plant Samples Collection and Processing

One month after inoculation, tomato roots were harvested from GDW1 inoculated and control plants according to the methodology of Hanifah [19]. For sample collection, roots were collected from five tomato plants per replicate from each treatment and thoroughly mixed to make one composite sample (a minimum of three biological replicates were collected from each group). To avoid contamination, the tomato roots were first surface sterilized with 75% ethanol for 60 s, followed by three-time washing with sdH_2_O, and then quickly dried with sterilized filter paper to absorb the excess water. Two sets of root samples (one for transcriptome study and one for host bacteriome study) were collected from each treatment and placed in liquid nitrogen until delivery to the laboratory. The root samples collected from CK and T were labeled CK (CK-1, CK-2, and CK-3) and T (T-1, T-2, and T-3) and then stored at −80 °C for transcriptome and 16S high-throughput amplicon sequencing.

### 4.6. Transcriptome Analysis

#### 4.6.1. Total RNA Extraction and Library Preparation

Plant total RNA (three biological replicates per treatment) was extracted using the RNeasy Mini Kit (Qiagen, Hilden, Germany) according to the manufacturer’s instructions. The RNA purity and integrity were assessed using an Agilent Bioanalyzer 2100 (Agilent Technologies, Santa Clara, CA, USA), and the RNAs having RNA integrity numbers (RINs) > 7 were selected to form the library [67]. Library preparation was conducted utilizing a TruSeq RNA poly-A selection kit (Illumina, San Diego, CA, USA) according to standard protocols and sequenced on an Illumina NovaSeq X plus platform at Guangdong Magigene Biotechnology Co., Ltd. (Guangzhou, China).

#### 4.6.2. Data Processing and Analysis of Differentially Expressed Genes

The raw data collected from the Illumina NovaSeq X plus platform were first subjected to quality control using fastp to obtain clean reads [68]. The clean reads were then annotated against the reference genome (*Solanum lycopersicum* Heinz 1706, GCA_000188115.4) using the HISAT2 tool with default parameters, and FPKM gene expression was estimated as values [69]. After obtaining the gene expression matrix of each sample, the DESeq2 software was used for differentially expressed gene (DEG) expression at a threshold level of adj.*p*.value < 0.05 and |Log2FC| > 0.5 [70]. PCA analysis was performed using the PCAtools package in R (v4.2.1). KEGG and GO enrichment analyses were performed using the clusterProfiler software package in R [71], and the species annotation files used were generated by the AnnotationHub using the ensembldb package in R [72]. The differential gene protein–protein interaction (PPI) network was constructed using the STRING (https://string-db.org) website, with a confidence level of 0.2 and the species *Solanum lycopersicum* [73]. Five algorithms, maximal clique centrality (MCC), maximum neighborhood component (MNC), betweenness, closeness, and degree, were used in the Cytoscape using the cytoHubba plugin for hub gene analysis [74]. The intersection gene analysis of the five algorithms was performed using the UpSetR package in R v.4.4.1 [75].

### 4.7. Analysis of the Tomato Plant Root Bacteriome Using 16S Amplicon Sequencing

#### 4.7.1. Total Genomic DNA Extraction and Library Construction

Tomato plant total genomic DNA was extracted from 0.5 g of each root sample using DNeasy Plant Kits by QIAGEN following the manufacturer’s protocols. The purity of the isolated DNA was measured using the OD_260/280_ ratio 1.7–1.9 via a spectrophotometer (NanoDrop 2000; Thermo Scientific, Waltham, MA, USA). PCR was amplified for the V5–V7 variable regions of the 16S gene of the bacteria using the primers 799F (5′-AACMGGATTAGATACCCKG-3′) and 1193R (5′-ACGTCATCCCCACCTTCC-3′) [76], which were subsequently sequenced on an Illumina MiSeq platform at Gene Denovo Biotechnology Co., Ltd. (Guangzhou, China) to obtain pair-end reads.

#### 4.7.2. Sequencing Data Processing and Bioinformatics Analyses

QIIME2 was utilized for quality control to remove primer sequences, chimeras, and low-quality reads with Q-scores < 30 [77]. DADA2 was employed to determine amplicon sequence variations (ASVs) [78]. ASVs were taxonomically assigned in the RDP database using the naïve Bayesian algorithm [79]. The alpha diversity dilution curve, alpha diversity index, and Euclidean distance matrix were analyzed using the corresponding plugins provided by the QIIME2 platform. The edgeR package in R was used to conduct differential bacteriome analysis between the T and CK groups; the differential screening conditions were *p* < 0.05 and |Log2FC| > 1 [80]. FAPROTAX function predictions were generated using functions in the microeco package in R [81], and KEGG level 2 functions were generated using the PICRUSt2 software [82]. Co-occurrence network analysis was conducted at the ASV level by eliminating the ASVs with relative abundances of less than 0.005 using the trans_network function in the microeco package in R v.4.4.1. Spearman correlation (*p* < 0.05) was used for network correlation, and Gephi 0.9.2 was used for visualization of the control group and treatment group network [83].

### 4.8. Different Joint Analyses

The Hmisc package in R was used to calculate the Spearman correlation coefficient between hub genes and ASVs at a threshold level (*p* < 0.05, |R| > 0.8). The correlation heatmap was generated using the corrplot package, and the scatter plot was generated using ggplot in R. The The Score_ASV-Hub_gene pathway regulatory network was visualized using Cytoscape [84], and Mantel.test analysis was performed via the ggcor package for calculation and visualization of correlations between ASV scores, hub genes, and plant growth parameters.

### 4.9. Validation of RNA-Seq Data by RT-qPCR and Statistical Analysis

RT-qPCR was amplified for the nine hub genes to validate RNA-seq data. The gene expression levels of the target genes were normalized using the tomato actin (*ACT*) gene as an internal reference according to the 2^−ΔΔCT^ method [85]. Three biological replicates were used for each gene. All the data are presented as mean (±SD). The data were statistically analyzed using analysis of variance (ANOVA), and to evaluate the significant differences between the means of data, Student’s *t*-test was used at the *p* < 0.05 level. All the graphs were merged and combined using Adobe Illustrator 2022.

## 5. Conclusions

**Figure 10:**
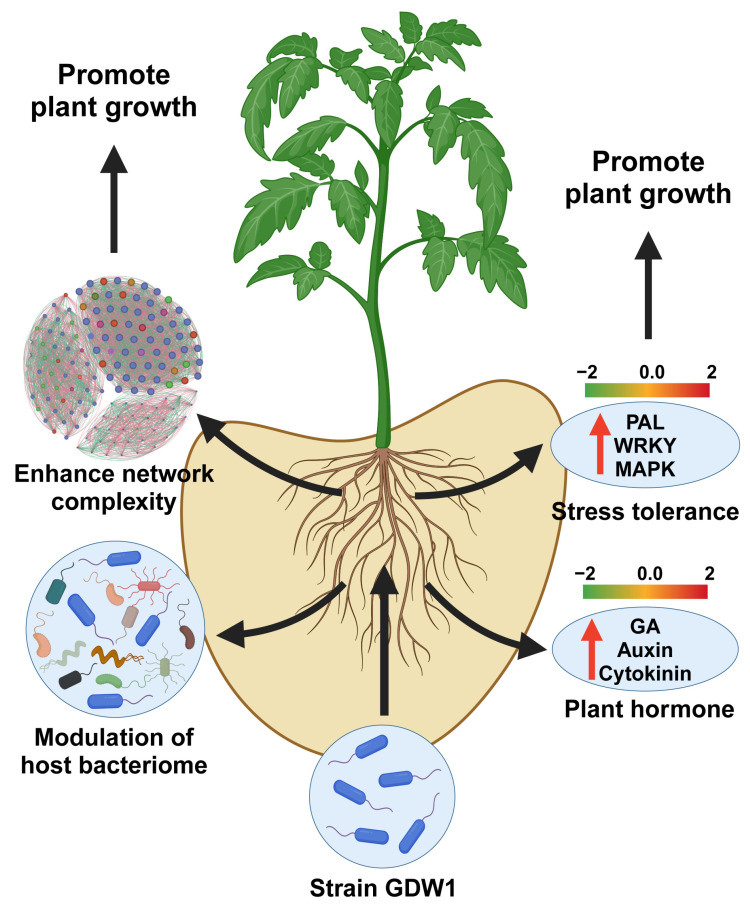
Underlying plant-growth-promoting mechanism of *Pseudomonas oryzihabitans* GDW1 in tomato. *P. oryzihabitans* GDW1 promotes tomato growth via modulation of the host root bacteriome, increasing network complexity by reducing competition for resources and inducing the expression of plant hormones and stress tolerance-related genes. The red arrows show the upregulated genes involved in plant hormone signal transduction and stress resistance. The figure was created using Adobe Illustrator 2022 and the online tool Biorender (https://www.biorender.com/).

In conclusion, this study provides compelling evidence for the plant-growth-promoting potential of *Pseudomonas oryzihabitans* GDW1, an endophytic bacterial strain isolated from a healthy pine tree, on tomato plants. Through a comprehensive multiomics approach, we elucidated the mechanisms by which GDW1 enhances tomato growth, modulates the host transcriptome, and reshapes the root bacteriome (Figure 10). The application of *P. oryzihabitans* GDW1 resulted in significant improvements in tomato plant growth parameters, including shoot length, root length, and biomass accumulation, highlighting its potential as a biofertilizer in sustainable agriculture. Transcriptomic analysis revealed 1158 differentially expressed genes in GDW1-treated plants enriched in plant hormone signaling pathways (auxin, gibberellin, cytokinin) and stress responses. Furthermore, 16S rRNA gene amplicon sequencing revealed that GDW1 inoculation altered the root bacterial community structure, increasing the diversity and abundance of beneficial taxa, particularly Proteobacteria and Bacteroidota. Co-occurrence network analysis indicated that GDW1 fostered a more complex and interconnected microbial network, potentially improving nutrient acquisition and plant health. Functional prediction analysis further suggested involvement of *P. oryzihabitans* GDW1 in nitrogen cycling, organic matter degradation, and stress resilience. These findings underscore the potential of *P. oryzihabitans* GDW1 as a sustainable alternative to chemical fertilizers, offering a promising strategy to enhance crop productivity while reducing reliance on agrochemicals. Future research should explore its application in field conditions and its potential to mitigate biotic and abiotic stresses in other crops.

## Figures and Tables

**Table 1:** Statistical analysis of clean reads mapping into reference genome of *Solanum lycopersicum*.

Sample	Total Reads	Clean Reads	Total Mapped Reads	Mapped Reads (%)	Q20 (%)	Q30 (%)	GC Content (%)	Clean Data Ratio (%)
CK.1	52,713,686	44,499,896	43,203,060	97.09	98.49	94.4	44.58	90.7
CK.2	53,326,478	46,835,834	45,637,248	97.44	98.53	94.47	44.12	91.42
CK.3	46,567,128	41,096,730	40,138,662	97.67	98.59	94.65	44.38	91.77
T.1	49,919,330	44,373,886	43,353,271	97.7	98.73	95.04	44.26	92.36
T.2	47,267,046	38,240,942	37,364,930	97.71	98.69	94.95	45.37	92.4
T.3	54,946,518	42,801,358	41,649,310	97.31	98.57	94.61	45.43	90.91
Total	304,740,186	257,848,646	251,346,481	97.49	98.6	94.69	44.69	91.59

Here: T: application of *P. oryzihabitans* GDW1; CK: application of water as control. CK.1, CK.2, CK.3, T.1, T.2, and T.3 are replicates per treatment, and each replicate contained the roots of five tomato plants.

## Data Availability

The data sets generated for this study can be found in the NCBI public database. All 16S rRNA gene and transcriptome analysis raw sequences can be found in Sequence Read Archive (SRA) under BioProject no. PRJNA1186330 and PRJNA1186314.

## Supplementary Materials

The following supporting information can be downloaded at: https://www.mdpi.com/article/10.3390/ijms26051922/s1.
